# History of drinking problems diminishes the protective effects of within-guideline drinking on 18-year risk of dementia and CIND

**DOI:** 10.1186/s12889-021-12358-4

**Published:** 2021-12-23

**Authors:** Penny L. Brennan, Charles J. Holahan, Rudolf H. Moos, Kathleen K. Schutte

**Affiliations:** 1 Institute for Health & Aging, University of California, San Francisco, Box 0646, 490 Illinois St., Floor 12, San Francisco, CA 94143 USA; 2 Department of Psychology, University of Texas at Austin, Austin, TX USA; 3 Department of Psychiatry and Behavioral Sciences, Stanford University Medical Center, Stanford, CA USA; 4 Independent Researcher, Cupertino, CA USA

**Keywords:** Older adults, Alcohol consumption, Drinking problems, Dementia, CIND

## Abstract

**Objective:**

To examine the moderating effect of older adults’ history of drinking problems on the relationship between their baseline alcohol consumption and risk of dementia and cognitive impairment, no dementia (CIND) 18 years later.

**Method:**

A longitudinal Health and Retirement Study cohort (*n* = 4421) was analyzed to demonstrate how older adults’ baseline membership in one of six drinking categories (non-drinker, within-guideline drinker, and outside-guideline drinker groups, divided to reflect absence or presence of a history of drinking problems) predicts dementia and CIND 18 years later.

**Results:**

Among participants with no history of drinking problems, 13% of non-drinkers, 5% of within-guideline drinkers, and 9% of outside-guideline drinkers were classified as having dementia 18-years later. Among those with a history of drinking problems, 14% of non-drinkers, 9% of within-guideline drinkers, and 7% of outside-guideline drinkers were classified with dementia. With Non-Drinker, No HDP as reference category, being a baseline within-guideline drinker with no history of drinking problems reduced the likelihood of dementia 18 years later by 45%, independent of baseline demographic and health characteristics; being a baseline within-guideline drinker with a history of drinking problems reduced the likelihood by only 13% (n.s.). Similar patterns obtained for the prediction of CIND.

**Conclusions:**

For older adults, consuming alcohol at levels within validated guidelines for low-risk drinking may offer moderate long-term protection from dementia and CIND, but this effect is diminished by having a history of drinking problems. Efforts to predict and prevent dementia and CIND should focus on older adults’ history of drinking problems in addition to how much alcohol they consume.

## Introduction

Almost 6 million Americans live with Alzheimer’s Disease and related dementias (ADRD), and more than one-third of U.S. adults over age 70 experience cognitive difficulties that fall short of dementia but significantly interfere with their daily functioning (cognitive impairment, but no dementia (CIND)) [1–4]. Prevalence of ADRD and CIND are expected to expand dramatically in the next several decades, fueled by the aging and size of post-World War II “baby boom” cohorts. This will diminish the length and quality of life of older adults, the health and well-being of their care providers, and expand costs associated with older adults’ health care [1, 5]. Thus, it is a public health priority to identify potentially modifiable predictors of ADRD and CIND in late life [6–10].

Older adults’ use of alcohol is an important, potentially modifiable risk factor for ADRD and CIND [6–15]. Research in this area has developed along two separate lines: One has focused on the association between the *quantity of alcohol* that older adults consume and the degree of their cognitive impairment. Although findings in this area are not completely consistent, they suggest overall that there is a “U”- or “inverted J” - shaped relationship between how much alcohol older adults consume and their subsequent risk of dementia and impaired cognitive function, wherein risk is least among “moderate” drinkers, and highest among non- and “heavy” drinkers [6–8, 12–14, 16–19].

A second, smaller line of research has focused on the relationship between having a *history of drinking problems* and subsequent risk of dementia or CIND. This line of inquiry is important to pursue because drinking problems or symptoms of alcohol use disorder (i.e., negative physical, psychological, and social consequences of alcohol use) are not synonymous with heavier alcohol consumption. In general, drinking problems are positively associated with higher amounts of alcohol consumption [20, 21] but these associations are often moderate, highlighting the fact that it is possible to have drinking problems even in the context of low-level alcohol consumption and the converse: people can suffer few or no apparent negative consequences of alcohol use despite consuming large quantities of alcohol. Further, having drinking problems may be indicative of using alcohol in ways that, cumulatively over the adult life course, result in metabolic changes, nutritional deficiencies, and other central nervous system changes linked to elevated risk of dementia and CIND [10, 11, 22, 23]. In support of this idea, two studies of older adults reported positive associations between having a history of drinking problems and later, lower cognitive function scores, over intervals ranging from 6 to 18 years [10, 11]. However, another study of older adults [17] found neither cross-sectional nor 3-year prospective associations between affirming a history of drinking problems and cognitive function scores.

Several factors limit the interpretability of findings from both of these lines of research. Definitions of “light”, “moderate”, and “heavy” drinking have varied widely among studies of older adults’ alcohol consumption and cognitive function outcomes [6, 8, 14, 24]. Moreover, most studies in this area have used cross-sectional or short-term follow-up designs. For example, of the 30 studies reviewed by Beydoun and colleagues [9], fewer than half were longitudinal and these had an average length of about 8 years. This is problematic because short follow-up intervals may prevent the detection of cognitive decline and dementia onset among older adults, especially if they cover periods very early, or very late, in the later life-span [8, 13].

This study seeks to overcome these limitations in several ways. First, we focus on amounts of alcohol consumption that are within and outside validated clinical and public health guidelines for low risk levels of alcohol consumption by men and by women, rather than by inconsistently defined “light”, “moderate”, and “heavy” amounts of alcohol consumption. Second, we examine the relationship between baseline alcohol consumption and subsequent dementia and CIND risk over a long time interval, beginning with when study participants are about age 60, and extending for the next 18 years. Finally, rather than separately examining the influence of amount of alcohol consumption and history of drinking problems on subsequent cognitive function, we will integrate these effects in our predictive models. Specifically, we will determine whether having a history of drinking problems has a moderating influence on the inverted-J or U-shaped relationship often found to describe the relationship between how much alcohol older adults consume and their cognitive function outcomes.

Based on the prior literature, we expect to find, among older adults *with no history of drinking problems*, an inverted-J or U-shaped relationship, wherein the prevalence of dementia and CIND will be highest among non-drinkers and outside-guideline drinkers, and lowest among within-guideline drinkers. We predict that, among older adults *with a history of drinking problems*, these inverted-J or U-shared curves will be *amplified*: non-drinkers, within-guideline drinkers, and outside-guideline drinkers will all have elevated prevalence of dementia and CIND compared to their counterparts with no history of drinking problems. We will determine whether these relationships hold independent of participants’ baseline demographic and health characteristics because these are important confounding factors known to influence both older adults’ use of alcohol and their risk of cognitive impairment [7, 13].

## Method

### Sample

The sample was drawn from the parent Health and Retirement Study (HRS), which has conducted biennial assessments of the health and economic characteristics of adults age 50+ since 1992. HRS longitudinal data have high follow-up rates and few missing data [25, 26].

Because cross-wave item content of HRS alcohol use measures did not become identical until 1996, this is the earliest available baseline assessment point for longitudinal study of HRS participants’ alcohol consumption [27, 28]. The HRS sample for this investigation (*n* = 4412) comprises HRS participants age 55 to 65 who completed the HRS 1996 core interview, survived 18 years, and provided self- or proxy-reported information about their cognitive function in the HRS 2014 core interview. This longitudinal sample consisted of about 50% of the participants who completed the baseline 1996 HRS core interview. At baseline 1996 assessment, participants who would die or be unable to fully participate in the 2014 HRS core interview were somewhat more likely to be male (*X*^*2*^(18229) = 64.6, *p* < .01), non-white (*X*^*2*^(18229) = 24.0, *p* < .01), older (F(1,8227) = 93.6, *p* < .01), less educated (F(1,8223) = 101.2, *p* < .01)), lower income (F(1,7678) = 118.4, *p* < .01), and less healthy, as indicated by having more medical conditions (F(1,8227) = 427.3, *p* < .01) and more depressive symptoms (F(1,8227) = 68.8, *p* < .01), than were the members of our 18-year HRS longitudinal sample. They were also somewhat more likely to smoke (*X*^*2*^(18229) = 198.3, *p* < .01), have a history of drinking problems (F(1,4129) = 49.9, *p* <. 01), and to consume more drinks per week (F(1,4129) = 12.9, *p* < .01).

### Measures

#### Demographic characteristics

Included baseline *age*, *sex* (female = 1, male = 0), *race* (white = 1, non-white = 0), years of *education*, and *family income*. We indexed socioeconomic status (*SES*) as the average of a participant’s years of education and current annual family income, using standard scores for both measures to equate their scales [29].

#### Health characteristics

Included baseline *smoking status* (yes = 1, no = 0), body mass index (*BMI*), and *medical conditions*, a count of 8 possible diagnosed medical conditions (e.g., cancer, diabetes), and *depressive symptoms*, assessed with the CES-D [30]. Cronbach’s alpha for this scale was .79. The HRS CES-D has good construct validity [31].

#### Drinking characteristics

In HRS interviews, *amount of alcohol consumed* is assessed with two questions: “In the last 3 months, on days you drank, about how many drinks did you have?” and “In the last 3 months, on average, how many days per week have you had any alcohol to drink (For example, beer, wine, or any drink containing liquor)?”. We used participants’ responses to these items to determine whether or not they met (*“within-guideline”*) or exceeded (*“outside-guideline”*) validated and widely used clinical and public health guidelines [32–36] for drinking at *low risk of developing alcohol use disorders.* These guidelines for women are: < 3 drinks per day *and* < 7 drinks per week; for men they are < 4 drinks per day *and* < 14 drinks per week. *Non-drinkers* were HRS participants who had not consumed alcohol in the last 3 months.

HRS participants’ *history of drinking problems* is assessed *only at their initial interview, at entry to the HRS study*, using the CAGE measure [37, 38], a screening tool used to distinguish between individuals, including older adults, with and without drinking problems [39, 40]. CAGE items comprise participants’ responses to four questions: “Have you ever felt that you should *cut down* on drinking?”, “Have people ever *annoyed* you by criticizing your drinking?”; “Have you ever felt bad or *guilty* about drinking?”, and “Have you ever taken a drink first thing in the morning (‘*eyeopener’*) to steady your nerves or get rid of a hangover?”. Because HRS participants’ CAGE responses were skewed toward having no history of drinking problems, we created a dichotomous *history of drinking problems* variable (0 = no; 1 = yes) indicating occurrence of one or more of the CAGE experiences at some point in life.

#### Cognitive function at 18-year follow-up

We assessed participants’ 2014 cognitive function using the Langa-Weir Classification of Cognitive Function [3]. This measure uses both HRS self-reported, and HRS proxy-reported, 2014 cognitive function information. HRS self-respondents (94% of the sample) were assessed with a 27-point cognitive function scale, adapted from the Telephone Interview for Cognitive Status (TICS) [41, 42]. Based upon relationships between TICS and HRS ADAMS cognitive function scores [2], three levels of cognitive function were defined: Normal (12–27 points), Cognitively Impaired but not Demented (CIND) (7–11 points), and Demented (0–6 points).

To include the cognitive function data of HRS participants unable to self-respond to the 27-point cognitive function test (6% of the sample), Langa and colleagues [2, 43] developed an 11-point cognitive function scale comprised of items assessing: (1) participants’ performance of instrumental activities of daily living, and their memory (2) as judged by a proxy respondent (e.g., spouse or child), and (3) as judged by the HRS interviewer. Cut-points for this cognitive function scale were: Normal (0–2 points), CIND (3–5 points), and Demented (6–11 points). Using a participant’s self-response *or* proxy cognitive function classification, which are mutually exclusive categories in the data, Langer and colleagues calculate an overall *summary classification,* the Langa-Weir Classification of *Normal, CIND, or Demented* cognitive function status. Participants fall into only one of these categories.

### Statistical analyses

Data were analyzed using descriptive statistics and multinomial logistic regression analyses. Multinomial logistic regressions that have both a categorical predictor with three or more levels, and a categorical outcome with three or more levels, have two reference groups: one pertaining to the predictive variable and one pertaining to the outcome variable. In this investigation, the reference group for the predictive drinking group variable was non-drinkers with no history of drinking problems. For the outcome variable it was cognitive function labeled “normal” according to the Langa-Weir classification scheme. All methods used in this investigation were performed in accordance with relevant guidelines and regulations.

## Results

### Demographic, health, and drinking characteristics at baseline in 1996

At baseline assessment in 1996, participants were on average about 60 years old; the sample was 59% female, and 82% white. Participants had completed an average of 12.5 years of education, and had an average household income of about $100,000 per year (Table 1). About 15% of participants smoked and had, on average, one diagnosed medical condition. Participants’ average BMI was about 27, mid-range overweight [44]. On average, participants had at least one depressive symptom, such as feeling slowed down, or having trouble sleeping.

**Table 1:** Baseline (1996) demographic, health, and drinking characteristics in overall sample and by drinking group

	Overall sample	Non-drinkers (*n* = 2052)		Within-guideline drinkers (*n* = 2113)		Outside-guideline drinkers (*n* = 247)			
		No HDP	Yes HDP	No HDP	Yes HDP	No HDP	Yes HDP	F/X^2^	*p*-value
*Demographic Characteristics*									
Age (M)	59.5	59.6	59.3	59.5	59.4	59.5	59.3	1.01	.410
Sex (% female)	59.0	73.0	38.0	57.0	30.0	63.0	40.0	84.4	.000
Race (% white)	81.9	77.2	72.7	87.6	82.0	89.2	88.3	18.0	.000
Education (M, yrs)	12.5	11.7	11.5	13.2	12.9	12.8	12.8	49.2	.000
Income (in thousands)	108.1	75.9	81.1	133.9	142.7	148.0	120.2	31.2	.000
*Health Characteristics*									
Smoking	15.2	12.8	17.7	13.7	20.4	27.4	31.0	12.54	.000
Medical conditions	1.1	1.2	1.2	1.0	1.0	1.1	1.0	12.4	.000
BMI	27.3	27.8	28.1	26.9	27.2	26.4	26.6	9.48	.000
Depressive symptoms	0.9	1.1	1.3	0.7	0.9	0.8	1.0	12.7	.000
*Drinking Characteristics*									
N of drinking problems (M)	0.4	0.0	2.2	0.0	1.6	0.0	1.7	4776.1	.000
N of drinks (per occasion) (M)	1.7	0.0	0.0	1.3	1.6	3.3	4.3	424.2	.000
N of drinks (total per week) (M)	4.8	0.0	0.0	2.4	4.4	17.1	24.2	756.7	.000

Note: *HDP* History of Drinking Problems, *M* mean, *%* percentage

At baseline, 21% of the overall sample affirmed one or more of the CAGE items used to assess presence of some history of drinking problems. Of these participants, 31% affirmed only the “cut down” item; 17% affirmed only one of the other three CAGE items; 26% affirmed two of the four items; and 26% affirmed three or more of the items (not shown).

At baseline, participants had an average of 0.40 current or past drinking problems, as assessed by the CAGE, consumed just over 1.5 drinks per drinking occasion, and drank a total of about 5 drinks per week (Table 1). About 47% of participants were Non-Drinkers, 48% were Within-Guideline Drinkers, and about 6% were Outside-Guideline Drinkers. Among Non-Drinkers, about 16% had a history of drinking problems; this was true of 21% of Within-Guideline Drinkers and almost 59% of Outside-Guideline drinkers.

### Predicting 18-year dementia from baseline drinking group

**Fig. 1:**
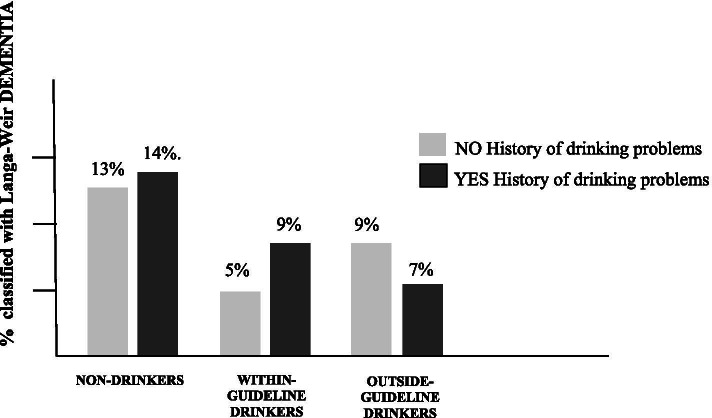
Percentage participants classified with dementia at 18-year follow-up, by baseline 1996 drinking group

According to the Langer-Weir Classification criteria, at the 18-year follow-up almost 70% of the overall sample had Normal cognitive function, 9% had Dementia, and 21% had CIND. Figure 1 shows the percentage of participants classified with Dementia at the 18-year follow-up, by 1996 drinking group classification. Among participants with *no history of drinking problems* (grey shading), about 13% of Non-Drinkers, 5% of Within-Guideline Drinkers, and 9% of Outside-Guideline Drinkers were classified as having dementia. Among participants *with a history of drinking problems* (black shading), 14% of Non-Drinkers, 9% of Within-Guideline Drinkers, and 7% of Outside-Guideline Drinkers were classified as having dementia.

The apparent protective effect of within-guideline drinking at baseline on risk of dementia 18 years later is statistically significant (top half of Table 2). In a predictive model unadjusted for baseline demographic and health characteristics, within-guideline drinkers with no history of drinking problems were 67% less likely than baseline non-drinkers with no history of drinking problems to be classified as having dementia 18 years later. Within-guideline drinkers *with* a history of drinking problems were 37% less likely to be classified as having dementia. In this model, the protective effect of drinking also included outside-guideline drinkers with a history of drinking problems: they were 50% less likely than non-drinkers with no drinking-problem history to be classified as having dementia 18 years later.

**Table 2:** Multinomial regressions predicting Langa-Weir classification of DEMENTIA and CIND at 18-year follow-up from baseline (1996) drinking group classification

	Unadjusted				Adjusted for demographic characteristics				Adjusted for demographic and health characteristics			
	OR	CI	W	*p*- value	OR	CI	W	*p*- value	OR	CI	W	*p*-value
**DEMENTIA**												
*Baseline Characteristics*												
*Drinking Group*												
ND: No HDP	_	_	_	_	_	_	_	_	_	_	_	_
ND: Yes HDP	1.27	(0.89, 1.80)	1.74	.19	1.27	(0.86, 1.86)	1.44	.23	1.20	(0.81, 1.77)	0.81	.369
WG: No HDP	**0.33**	(0.26, 0.43)	67.6	.00	**0.52**	(0.40, 0.69)	20.97	.00	**0.55**	(.041, .073)	17.34	.000
WG: Yes HDP	**0.63**	(0.44, 0.91)	06.2	.01	0.91	(0.61, 1.35)	0.23	.63	0.87	(0.58, 1.30)	0.48	.490
OG: No HDP	0.61	(0.30, 1.24)	1.88	.17	1.01	(0.48, 2.13)	0.00	.98	1.00	(0.47, 2.12)	0.00	.994
OG: Yes HDP	**0.50**	(0.26, 0.98)	4.13	.04	0.77	(0.38, 1.56)	0.52	.47	0.73	(0.36, 1.47)	0.79	.375
*Demographic*												
Age					**1.14**	(1.10, 1.19)	52.91	.00	**1.15**	(1.11, 1.19)	54.33	.000
Female					0.95	(0.74, 1.20)	0.20	.66	0.89	(0.69, 1.14)	0.87	.351
White					**0.34**	(0.26, 0.43)	74.74	.00	**0.36**	(0.28,0.46)	63.86	.000
SES					**0.28**	(0.23, 0.33)	190.62	.00	**0.30**	(0.25, 0.36)	155.23	.000
*Health*												
N medical conditions									**1.24**	(1.11, 1.38)	15.19	.000
Smoking									**1.50**	(1.11, 2.02)	7.14	.008
BMI									1.02	(1.00, 1.04)	2.75	.097
Depression									1.07	(0.99, 1.14)	3.32	.069
**CIND**												
*Baseline Characteristics*												
*Drinking Group*												
ND: No HDP	_	_	_	_	_	_	_	_	_	_	_	_
ND: Yes HDP	**1.41**	(1.08, 1.85)	06.4	.01	1.33	(0.99, 1.78)	3.69	.055	1.26	(0.94, 1.69)	2.40	.121
WG: No HDP	**0.57**	(0.48, 0.67)	42.3	.00	**0.78**	(0.65, 0.94)	6.87	.009	**0.79**	(.065, .095)	6.28	.012
WG: Yes HDP	0.78	(0.60, 1.00)	03.6	.06	0.98	(0.74, 1.30)	0.02	.891	0.95	(0.71, 1.26)	0.14	.706
OG: No HDP	0.68	(0.40, 1.13)	2.23	.14	0.97	(0.56, 1.67)	0.01	.914	0.89	(0.51, 1.57)	0.15	.695
OG: Yes HDP	0.92	(0.61, 1.37)	0.17	.68	1.21	(0.79, 1.86)	0.75	.385	1.08	(0.70, 1.68)	0.13	.719
*Demographic*												
Age					**1.08**	(1.05, 1.10)	31.30	.000	**1.08**	(1.05, 1.11)	35.0	.000
Female					**0.83**	(0.70, 0.98)	5.06	.024	**0.79**	(0.66, 0.93)	7.79	.005
White					**0.36**	(0.30, 0.44)	113.31	.000	**0.39**	(0.32, 0.47)	96.83	.000
SES					**0.41**	(0.36, 0.47)	195.95	.000	**0.44**	(0.39, 0.50)	159.36	.000
*Health*												
N medical conditions									**1.16**	(1.07, 1.26)	13.70	.000
Smoking									**1.41**	(1.14, 1.75)	10.24	.001
BMI									1.01	(1.00, 1.03)	02.55	.111
Depression									**1.09**	(1.03, 1.14)	10.17	.001
-Log-Likelihood	68.96 (X2 = 133.86, df = 10, *p* = .000)				464.02 (X2 = 811.73, df = 18, *p* = .000)				6118.11 (X2 = 861.09, df = 26, *p* = .001)			
Nagelkerke Pseudo R2 = .	.037				.210				.225			

Note: *ND* Non-Drinker, *WG* Within-Guideline, *OG* Outside-Guideline, *OR* Odds ratio, *CI* Confidence interval, *W* Wald statistic

Independent of baseline demographic characteristics, within-guideline drinkers with no history of drinking problems reduced chances of dementia by 48%, whereas those with a history of drinking problems reduced them by only 9%. Independent of baseline demographic *and* health characteristics, within-guideline drinkers with no history of drinking problems were 45% less likely to have dementia; those with a history of drinking problems at baseline were only 13% less likely to be classified as having dementia, and this effect was not statistically significant. Any protective effect of drinking outside guidelines was diminished, and no longer statistically significant, once baseline demographic and health characteristics were statistically controlled.

### Predicting 18-year CIND from baseline drinking group

**Fig. 2:**
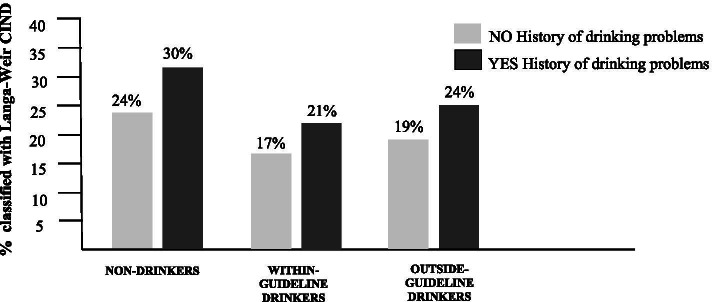
Percentage participants classified with CIND at 18-year follow-up, by baseline 1996 drinking group

Figure 2 shows the distribution of percentage participants with CIND classification by baseline drinking group. Among participants with *no history of drinking problems* (grey shading), 24% of Non-Drinkers, 17% of Within-Guideline Drinkers, and 19% of Outside-Guideline Drinkers were classified as having CIND. Among participants *with a history of drinking problems* (black shading), 30% of Non-Drinkers, 21% of Within-Guideline Drinkers, and 24% of Outside-Guideline Drinkers were classified with CIND.

The bottom half of Table 2 shows that, in an unadjusted predictive model, relative to non-drinkers with no history of drinking problems, non-drinkers *with* a history of drinking problems were 41% more likely to have CIND. Those who drank within-guidelines and had no history of drinking problems were 43% less likely to be classified with CIND.

After adjusting for baseline demographic and health characteristics, only within-guideline drinking, with no history of drinking problems, had a statistically significant effect on likelihood of CIND: It was a modest 21% reduction of risk of CIND.

## Discussion

Past research on the effects of older adults’ drinking on their risk of dementia and cognitive impairment has comprised two separate streams of research: one focused on average *quantity of alcohol consumed*, and another on *history of drinking problems*, as predictors of dementia and CIND. Here, we integrated these two approaches, showing how a history of drinking problems moderates average alcohol consumption in predicting cognitive impairment and dementia. First, among participants with no history of drinking problems, we highlighted the prospective association between within-guideline alcohol consumption and later enhanced cognitive function in aging. Next, we showed that having a history of drinking problems (i.e., consuming alcohol in ways that result in negative physical, psychological, or social consequences) diminishes the advantageous association between within-guideline alcohol use and cognitive function and can exacerbate the adverse association between outside-guideline drinking and cognitive impairment.

Building on previous research [6–8, 12–14, 16–19], we found — among participants with no history of drinking problems — a shallow inverted-J- or U-shaped relationships between baseline quantity of alcohol consumption and risk of dementia and CIND. That is, among older adults with no history of drinking problems, within-guideline drinkers, compared to non-drinkers and outside-guideline drinkers, showed reduced risk of dementia and CIND 18 years later.

Within-guideline alcohol consumption among individuals with no history of drinking problems may exert protective effects against dementia and CIND by elevating antioxidant and HDL lipoprotein levels [45] and through decreasing fibrinogen level and plasma viscosity [46]. This level of alcohol consumption may also increase brain-derived neurotrophic factor, which promotes brain plasticity and development [19]. More generally, an underlying association between within-guideline alcohol consumption and better overall cardiovascular health may, in part, explain enhanced cognitive functioning [46].

Extending previous research [10, 11], we demonstrated that a history of drinking problems altered the inverted-J and U-shaped relationships between the within-guideline alcohol consumption and enhanced cognitive function observed among individuals with no history of drinking problems. In the context of having a history of drinking problems, the associations of non-drinking and outside-guideline drinking with poorer cognitive function were generally amplified, and the relationship of within-guideline drinking to better cognitive function was diminished.

The mechanisms whereby within-guideline alcohol consumption may provide protection against cognitive loss appear to be counteracted by having a history of drinking problems. One reason may be that people with histories of drinking problems are more likely to engage in episodic heavy or binge drinking compared to those with no history of drinking problems [47, 48]. Concentrated, high-volume ethanol delivery to the brain can damage brain metabolism and morphology [45]. Excessive alcohol use results in under-nutrition of brain cells through metabolic changes and reduced folate and thiamine [6, 45]. In addition, high alcohol exposure causes neuroinflammation, neuron loss, reduced brain volume, and is linked to increased cardiovascular risks involving hypertension, atrial fibrillation, cardiomyopathy, and stroke, which may partially explain poorer cognitive function [6, 45].

High-volume ethanol delivery in earlier adulthood may have far-reaching consequences for late-life risk of dementia and CIND. Heavy and binge drinking in adolescence and early adulthood have been shown associated with alterations in brain morphology, neural functioning and structure, and performance on cognitive function tasks (e.g., [49, 50]) though it is not yet known whether or how alcohol-related changes in brain structure and function in earlier life influence subsequent risk of dementia and CIND for older adults.

This study had several strengths, including a large sample size, a longitudinal design featuring an 18-year follow-up interval, and use of validated classification systems to assess alcohol consumption levels and cognitive function outcomes. However, it also had limitations. The HRS measure of history of drinking problems is completed only once, when participants first enter the study, comprises only four items, and indicates only whether participants have “ever” experienced the items. Accordingly, it is not possible to infer the time of onset, duration, or severity of participants’ drinking problems over the life course. The outside-guideline drinking group was small, which may have diminished the likelihood of our detecting effects of membership in this group on cognitive function outcomes. Moreover, although our research design has the benefit of temporal precedence between predictive and outcome variables, it does not demonstrate a causal link between alcohol consumption and cognitive function. Although we controlled for key sociodemographic and health factors, there may be other variables associated with alcohol use and cognitive function that were uncontrolled and account for the associations we found between participants’ initial drinking behaviors and subsequent cognitive function. Finally, the 18-year longitudinal cohort studied here was subject to selective survival and other attrition processes that must be considered in interpreting the findings [51]. Our findings are generalizable only to younger-older adults, who survived 18 years, and from whom self-report or proxy-based cognitive function assessments could be obtained at follow-up. Moreover, selective survival and other attrition processes have likely masked the full impacts of alcohol use on cognitive function for members of the overall HRS 1996 study cohort. For example, over the 18-year span of this investigation, the costs of alcohol misuse for cognitive function among participants who were, at baseline in 1996, younger, sicker, and more disadvantaged educationally, economically, and with respect to drinking history, may have been considerable, but because these participants were more likely to be culled from our longitudinal sample due to death and physical or cognitive incapacitation, we cannot estimate those costs from our longitudinal sample data.

## Conclusions

For older adults, consuming alcohol at levels within validated guidelines for low-risk drinking may offer some long-term protection from dementia and CIND, but this effect is diminished by having a history of drinking problems. This finding has potential public health significance. It reinforces previous findings [52, 53] showing that low-risk drinking by older adults requires that they avoid both high levels of alcohol consumption *and* a signature drinking behavior associated with history of drinking problems, the practice of clustering many drinks within single drinking occasions, a behavior known to be increasing among older adults in the U.S. throughout the past decade [54, 55]. Clinical and public health efforts aimed at prediction and prevention of dementia and CIND may be improved by shifting emphasis from an exclusive focus on how much alcohol older adults consume to also include consideration of their problem-drinking histories and patterns of alcohol use.

## Data Availability

Data analyzed in this study were a re-analysis of existing data, which are openly available at the location cited in the Method and Reference sections of this manuscript, https://hrs.isr.umich.edu.

## Abbreviations

ADRD: Alzheimer’s Disease and related dementias
ADAMS: Aging, demographics, and memory study
BMI: Body mass index
CES-D: Center for Epidemiologic Studies Depression Scale
CIND: Cognitive impairment, no dementia
HDL: High-density lipoproteins
HDP: History of drinking problems
HRS: Health and Retirement Study
n.s.: Statistically non-significant
SES: Socioeconomic status
TICS: Telephone Interview for Cognitive Status
